# Inhalable Nanomedicines for Chronic Obstructive Pulmonary Disease: Recent Developments and Translational Barriers

**DOI:** 10.34172/apb.46836

**Published:** 2026-05-23

**Authors:** Ahmad Ainurofiq, Elizabeth Agrindha Rosalin, Mayra Shafwa Istiyono, Natasya Anggira Maharani, Ryan Fauzy, Salma Nurhalizah Sugiharti, Yuliana Fania Safa

**Affiliations:** ^1^Pharmaceutical Technology and Drug Delivery, Department of Pharmacy, Universitas Sebelas Maret, Ir. Sutami 36A, Surakarta, Indonesia, 57126; ^2^Department of Pharmacy, Universitas Sebelas Maret, Ir. Sutami 36A Surakarta, Indonesia, 57126

**Keywords:** COPD, Inhaled nanomedicine, Nanoparticles, Drug delivery, Pulmonary therapy

## Abstract

Chronic Obstructive Pulmonary Disease (COPD) is a condition that poses major challenges in therapeutic management, primarily due to the limitations of conventional inhalation methods in achieving optimal drug deposition and maintaining therapeutic effects over a long period. Inhalable nanomedicine has emerged as an alternative approach potentially utilizing nanoparticle-based drug delivery systems to potentially enhance pulmonary drug delivery. These systems may improve drug distribution within the lungs and reduce systemic exposure. Various platforms, including liposomes, solid lipid nanoparticles (SLN)/nanostructured lipid carriers (NLC), and polymeric nanoparticles, have demonstrated encouraging results in preclinical studies. However, the current evidence remains largely limited to in vitro and animal models, and their clinical relevance in COPD patients is not yet fully established. In addition, several challenges persist, including formulation stability, long-term safety, and scalability for large-scale production. Overall, while inhalable nanomedicine represents a promising strategy for improving COPD management, further well-designed clinical studies and translational research are required to validate its safety, efficacy, and applicability in clinical practice.

## Introduction

 Chronic Obstructive Pulmonary Disease (COPD) is one of the most common chronic respiratory disorders worldwide, and has been recognized as the third leading cause of death globally after ischemic heart disease and stroke.^1^ This disease is considered deadly because the process of airway obstruction is progressive and irreversible, causing decreased lung capacity, chronic hypoxia, respiratory failure, and systemic complications such as pulmonary hypertension and cardiovascular disease, which significantly increase the risk of mortality.^2^ This condition imposes a significant burden by reducing lung capacity, decreasing productivity, and increasing long-term care costs. Global epidemiology shows an uneven distribution of cases, with the highest prevalence in older populations in low- to middle-income countries exposed to tobacco smoke, air pollution, and biomass fuel. The 2020 Global Burden of Disease report estimates that COPD affects more than 200 million people worldwide, with 3.2 million deaths per year, underscoring its urgency as a public health issue. This trend indicates that without evidence-based prevention and management strategies, prevalence, mortality, and disability-adjusted life years (DALY) loss will continue to increase as the population ages and exposure to risk factors persists.

 In line with the high prevalence and health burden, conventional COPD therapies currently available, such as bronchodilators, inhaled corticosteroids, combination inhalers, and long-term oxygen therapy, still face various limitations. A clinical study showed that the combination of bronchodilators and inhaled corticosteroids can reduce the frequency of exacerbations, but the recurrence rate remains high.^3^ Other studies have shown that the use of triple combination inhaler therapy (ICS/LABA/LAMA) can improve lung function and increase the quality of life of patients, but is still accompanied by the risk of systemic side effects such as pneumonia.^4^ Similarly, a report confirms that long-term inhaled corticosteroids only provide moderate benefits in reducing exacerbations, but are accompanied by an increased risk of lung infection.^5^

 In addition, the use of single bronchodilators is often ineffective for patients with severe COPD because clinical responses vary greatly between individuals. The 2025 GOLD Report highlights that drug particle deposition in the airways is often uneven, especially in patients with severe obstruction, so most of the drug does not reach the distal areas of the lungs.^6^ Long-term oxygen therapy has been shown to improve survival in chronic hypoxemic COPD, but daily use is considered impractical, expensive, and can cause oxidative complications if not closely monitored. Therefore, although conventional therapy plays an important role in symptom control, limitations in bioavailability, drug distribution, systemic side effects, and patient compliance emphasize the need for innovative drug delivery strategies, one of which is through inhaled nanomedicine, which is expected to improve the effectiveness and safety of therapy.

 Nanotechnology has recently been widely used, especially in the field of medicine, or otherwise known as nanomedicine. Nanomedicine is a field in health that applies nanotechnology by utilizing the physical, chemical, and biological properties of medicinal substances on the nanometer scale.^7^ Conventional inhalation therapies such as aerosols cannot effectively reach the alveolar region because the large particle size causes the drug to accumulate in the upper respiratory tract, resulting in the desired dose not reaching the target. In addition, conventional inhalation therapy is easily lost from the lungs due to the mucociliary clearance and alveolar macrophage mechanisms, requiring repeated doses.^8^ Treatment using nano-modification will provide more optimal therapeutics. Nanotechnology will increase the surface area of the drug that comes into contact with the lungs, thereby increasing its interaction. Nanotechnology can be combined with polymers that make the system more targeted and increase the concentration of a drug in specific locations, thereby increasing drug bioavailability. This system provides the advantage of therapeutic delivery that avoids first-pass metabolism and reduces the risk of toxicity.^9^ Lipid-polymer nanoparticles have high stability in biological circulation, good biodegradability, and high encapsulation efficiency, thereby maintaining the stability of active compounds and increasing drug bioavailability at specific locations.^10^ Nanoliposomes are made of phospholipid components that are compatible with the lung surface, thereby minimizing local irritation. Liposomes can increase intracellular absorption, prolong drug release, and reduce the rate of pulmonary clearance.^11^

 Recent research on inhalable nanomedicine has shown rapid progress, particularly through preclinical and early clinical studies that demonstrate the potential of inhalable nanomedicine to improve targeted drug delivery to lung tissue in COPD. Inhalable nanomedicine platforms, including polymeric nanoparticles, liposomes, lipid-based carriers, nanogels, and nanocrystals, have shown promising preclinical results and some early clinical evidence supporting improved drug delivery to the deep lungs and the potential for reduced systemic side effects in COPD.^12^ Several clinical studies have also demonstrated the potential of inhalable nanomedicine to improve the effectiveness of therapy for several respiratory diseases, including COPD. However, translating these research results to the clinical level still faces significant challenges.^13^ Some studies are still limited to in vitro animal models, which often cannot represent the complexity of COPD pathophysiology in humans, such as chronic exposure to cigarette smoke, airway remodeling, and comorbidities.^12^ This is supported by other research showing that the clinical application of amikacin liposome inhalation suspension (ALIS) for pulmonary infectious diseases, even though it has reached the advanced trial phase and provides efficacy and a local and reversible side effect profile, remains limited; thus, the application of the nanocarrier concept in COPD therapy is still in its early stages with few COPD clinical trials and inconsistent results.^14^

 In addition, differences in administration routes, doses, and evaluation parameters between studies, making it difficult to predict the pharmacokinetic profile of an inhalable nanomedicine.^15^ Therefore, a more standardized approach is needed to highlight differences in preclinical models, emphasize the importance of robust inhalation pharmacokinetic/pharmacodynamic studies, and follow-up for translation (GLP toxicology, dose-scaling, inhalation FIH studies).^15^ Another challenge in the development of inhalable nanomedicine is the limitation of large-scale production and technology transfer from the laboratory to the industrial level (scale-up). Most nanoparticle manufacturing methods used at the research stage are designed for small scales and are not easily applied to sustainable industrial processes. In addition, cGMP regulations for inhalable nanomedicine remain non-standardized.^16^ This article will discuss the potential scale-up technologies and the application of Quality by Design (QbD) principles in scaling up from the laboratory to the pilot scale.

 Despite the rapid expansion of inhalable nanomedicine research, most existing reviews primarily focus on general pulmonary drug delivery systems or other respiratory diseases, such as lung cancer, infectious diseases, and cystic fibrosis, with limited attention on the disease-specific complexities of COPD.^12,13^ This represents a critical gap, as COPD exhibits distinct pathophysiological features including mucus hypersecretion, airway remodeling, chronic inflammation, and heterogeneous airflow limitation that directly influence nanoparticle deposition, retention, and therapeutic efficacy.^17^ Moreover, previous reviews often discuss nanocarrier systems and translational challenges separately, without systematically linking them to disease-specific barriers.^18^ As a result, there is still a lack of integrated understanding of how nanomedicine design can be optimized according to COPD pathophysiology while simultaneously addressing real-world translational constraints, such as scalability, regulatory requirements, and clinical applicability.

 Consequently, there remains a lack of integrated understanding of how nanomedicine design can be optimized according to COPD pathophysiology while simultaneously addressing real-world translational constraints, such as scalability, regulatory requirements, and clinical applicability.^12^ Therefore, this review aims to provide a critically integrated perspective by analyzing COPD-specific biological and physiological barriers to inhaled drug delivery, evaluating various nanocarrier platforms based on their ability to overcome these barriers, and examining key translational challenges that may limit clinical implementation. By bridging these aspects, this review offers a more insight-driven framework for the rational design and clinical translation of inhalable nanomedicine in COPD.

## Pathophysiology of COPD and Drug Delivery Barriers Changes in Lung Structure

###  Pathophysiology of COPD

 COPD is characterized by chronic changes in the structure and function of the lungs, including persistent inflammation, remodeling (structural changes) of the airways, and excessive mucus hypersecretion. Prolonged exposure to irritants, such as cigarette smoke and air pollution, is a major risk factor that can trigger the inflammatory process and cause damage to the alveoli and small airways.

 The lung structure consists of a fine branching network of airways known as the bronchial tree, ending in alveolar sacs where gas exchange occurs. The lungs have a sponge-like structure and consist of various types of immune cells that function in the body’s innate defense against foreign particles and help repair damaged tissue. Most pathological immune responses occur in the bronchial wall, which is lined with cilia and a layer of mucus secreted by goblet cells. This layer of mucus captures inhaled foreign particles and removes them continuously through the movement of the cilia.^17^

 The entry of foreign substances into the respiratory tract can trigger an excessive inflammatory response, resulting in smooth muscle contraction, mucus gland enlargement, and mucosal swelling. These conditions trigger chronic bronchitis, ciliary dysfunction, and bronchiolar narrowing.^9^ In addition, emphysema is also a major component of COPD. Exposure of alveolar epithelial cells (AECs) to irritants and oxidative molecules activates both the innate and adaptive immune systems. Once activated, AECs release cytokines, chemokines, and other mediators to regulate the immune response. At the same time, alveolar macrophages are triggered to produce proteolytic enzymes, including elastase and matrix metalloproteinases (MMPs), which can result in tissue damage. An imbalance in protease activity and increased apoptosis ultimately lead to the destruction of the alveolar structure. During the tissue repair phase, the collagen deposition process can actually worsen the condition because it causes a loss of alveolar elasticity, thus disrupting the lungs’ ability to contract and relax normally.^17^

###  Barriers to the Delivery of Inhaled Medications

 Structural and functional changes in the lungs of COPD patients can inhibit the effectiveness of inhalation therapy. The mucociliary clearance mechanism, which cleans foreign particles from the airways, becomes inefficient due to increased mucus viscosity and damage to ciliated cells. This condition causes inhaled drug particles to be trapped in the upper airways or even expelled before reaching the site of therapeutic action.^19^ Drug deposition is also limited due to narrowing of the bronchiolar lumen, changes in airflow patterns, and ventilation heterogeneity in damaged lungs, so that only a small portion of drug particles reach the alveoli while most are deposited in the upper airways and the concentration of drugs reaching the target site is low.^20^ Additionally, the permeability of lung tissue to drugs decreases due to epithelial changes such as wall thickening and subepithelial fibrosis, thereby inhibiting drug penetration into target tissues and reducing local absorption capacity, which ultimately diminishes the therapeutic effect of inhaled drugs.^21^ These limitations highlight the need for more advanced drug delivery strategies to improve therapeutic outcomes. In this context, nanotechnology-based inhalation systems have emerged as a promising approach to enhance drug deposition, stability, and targeted delivery within the lungs. The pathophysiological mechanisms underlying COPD are illustrated in Figure 1.

**Figure 1:**
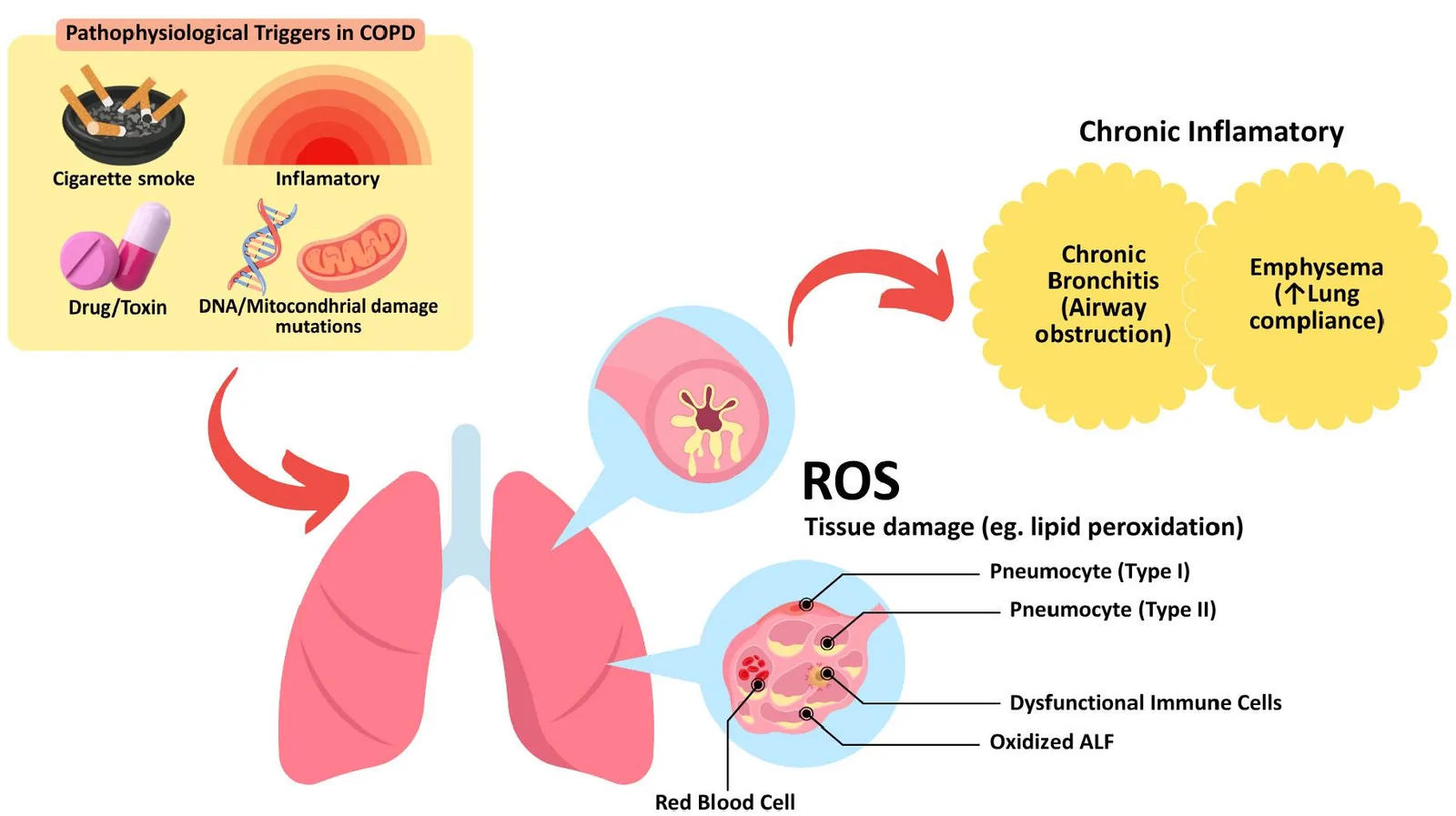
Pathophysiology of COPD

## Conventional Inhalation Therapy

###  Achievements and Limitations of Medicines

 The most commonly used treatment for COPD at present is conventional inhalation therapy. Conventional inhalation is chosen because it delivers medication directly to the lungs or airways, resulting in a rapid onset of action and local effects. The medications commonly used in inhalation therapy are bronchodilators, corticosteroids, or a combination of both to work synergistically.^22^ Conventional inhalation therapy is not only used to control symptoms but also to prevent exacerbations that can lead to decreased lung function and are the primary cause of mortality in COPD. Inhalation is recommended as the primary treatment for COPD due to its effectiveness in quickly relieving symptoms, preventing exacerbations, and its ease of use.^6^

 Bronchodilators are one type of therapy that can be used to treat COPD. Bronchodilators work by relieving chronic and progressive airway obstruction. Bronchodilators relax the smooth muscles of the bronchi, allowing the airways to widen and air to flow in and out smoothly again. Improved ventilation reduces the symptoms of shortness of breath.^23^ Bronchodilators are divided into two types based on their mechanism of action, namely β2-adrenergic agonists and muscarinic antagonists. β2-adrenergic agonists work by stimulating β2 receptors in the smooth muscles of the bronchi to increase intracellular cyclic adenosine monophosphate (cAMP) levels serum, causing the bronchial muscles to relax. Examples of β2-adrenergic agonists are salbutamol and formoterol. Muscarinic antagonists work by blocking muscarinic receptors, especially the M3 subtype, which inhibits acetylcholine-induced bronchoconstriction in the smooth muscles of the airways. Examples of muscarinic antagonists are ipratropium bromide and tiotropium.^24^

 Inhaled corticosteroids are drugs that function to control the chronic inflammatory process in the pathogenesis of COPD by inhibiting the activation of pro-inflammatory transcription factors such as nuclear factor-kappa B (NF-κB) and activator protein-1 (AP-1), which reduce the production of pro-inflammatory cytokines (such as IL-8, TNF-α, and IL-6), reducing neutrophil chemotaxis, and suppressing oxidative stress that plays a role in lung tissue damage. This mechanism suppresses chronic inflammation, reduces airway edema, and slows the progression of declining lung function. Examples of corticosteroids used for COPD are budesonide, fluticasone propionate, and beclomethasone dipropionate.^25^ Inhaled corticosteroids are not commonly used as monotherapy because their function is limited to pulmonary inflammation, not symptoms relief of shortness of breath. Therefore, inhaled corticosteroids are commonly combined with bronchodilators to provide a synergistic effect. The synergistic combination effect is the increased corticosteroid penetration due to bronchodilators-induced widening of the bronchial lumen.^26^

## Inhalation Device

###  Pressurized Metered Dose Inhaler (pMDI)

 The most commonly used respiratory drug delivery devices are pressurized metered dose inhalers (pMDIs), dry powder inhalers (DPIs), and nebulizers. A pressurized metered dose inhaler is an inhalation device that uses pressure to deliver medication into the respiratory tract in the form of an aerosol.^27^ This device has a measuring valve to regulate the amount of medication sprayed into the respiratory tract, ensuring a consistent dose with each use.^28^ The medication in a pMDI is in the form of a solution or suspension carried by a gas called a propellant. The solution or suspension form means that pMDIs often contain surfactants to prevent aggregation and phase separation between the solvent and the medication, resulting in stable medication release and stable medication.^29^ The aerodynamics within the pMDI are a very important component in delivering the drug. Aerodynamics will affect the particle size, morphology, and density of the drug. These factors will affect the deposition of the drug in the respiratory tract. The therapeutic efficacy of pMDIs is also influenced by the distribution of drug particles in the respiratory tract.^30^ The challenge in using pMDIs is the accuracy of their use. A study found that only 23% of patients who used pMDIs did so correctly, resulting in the treatment not being effective.^31^

###  Dry Powder Inhaler (DPI)

 A Dry Powder Inhaler (DPI) delivers medication to the lungs in dry-powder form. DPIs often face the challenge of achieving effective pulmonary drug delivery. The medication in the DPI is in the form of micronized powder measuring < 5 µm. The medication is delivered to the respiratory tract not by pressure assistance as in pMDI, but by the airflow from the patient’s own inspiration.^32^ A disadvantage of DPIs is the need for a sufficiently strong breath to distribute the medication into the respiratory tract, whereas it is known that patients with COPD often have respiratory problems that make it difficult to take deep breaths. A study showed that 10.8% of patients had difficulty using DPIs because it required deep inspiration.^33^ The primary challenge in dry powder inhaler (DPI) formulations is generating an aerosol with particles small enough to reach the lower airways. The effectiveness of respiratory medication delivery using DPIs is significantly affected by particle size and deagglomeration during the patient’s inspiratory flow. High humidity during storage promotes powder agglomeration, resulting in larger, clumped particles that are less likely to deposit effectively in the lungs.^34^

###  Nebulizer

 A nebulizer is a device that converts liquid into vapor. The vapor produced is channeled through a tube and exits through a mask-shaped nozzle, where it is inhaled by the patient. The aerosol or vapor produced contains active ingredients that help relieve symptoms caused by COPD.^35^ Unlike DPIs, a nebulizer does not require strong inspiration to draw the medication into the respiratory tract, making it more suitable for patients experiencing an asthma attack.^36^ The disadvantage of nebulizers is that they are unable to nebulize suspensions, whereas it is known that many active ingredients are water-insoluble and are therefore formulated in suspension form.^37^ In addition, nebulizers use heating, so active ingredients that are not heat-resistant will degrade due to heating.^38^ The three main inhalation devices used in clinical practice are illustrated in Figure 2.

**Figure 2:**
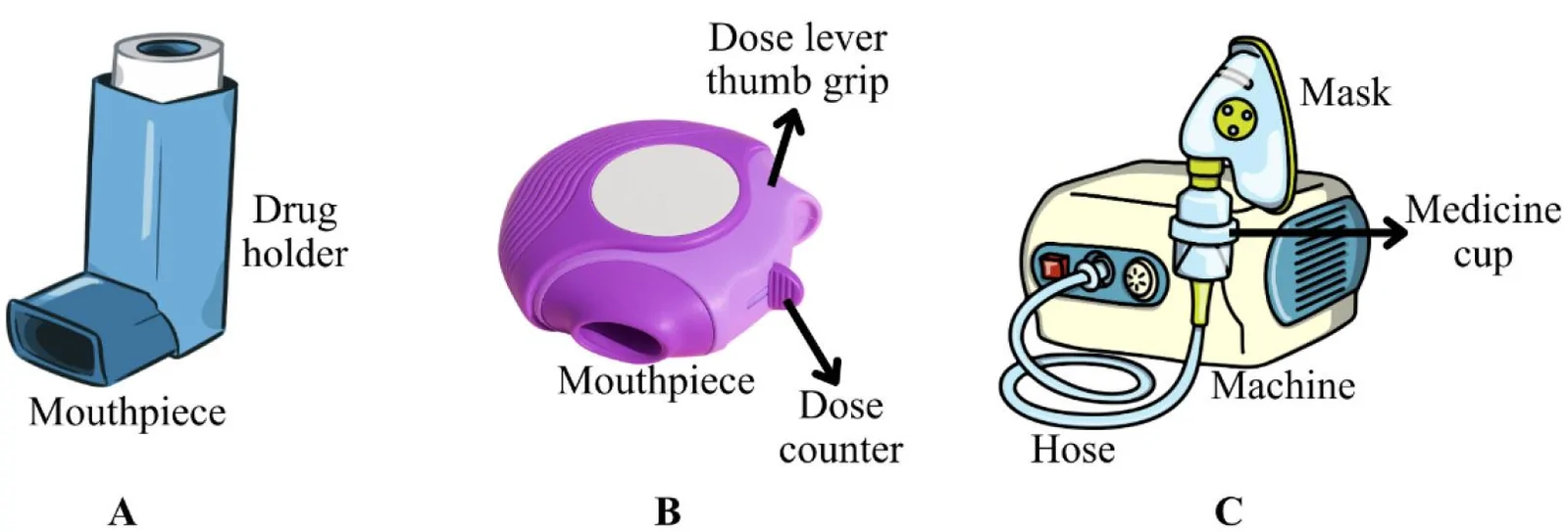
Illustration of inhalation devices used clinically to deliver medication to the lungs. The figure depicts three main classes of inhalation devices used in clinical practice: (A) Inhaler; (B) Dry Powder Inhaler (DPI); and (C) Nebulizer

## Limitations of Conventional Therapy

 The limitation of these drugs is their low local bioavailability due to suboptimal drug deposition in the lungs. Large particle size will result in suboptimal drug penetration. In addition, incorrect use of inhalers or nebulizers can also result in the drug not being distributed to the distal lung, which is where inflammation and obstruction most often occur. Large particle size can also affect the rate of drug clearance in the lungs. The mucociliary clearance mechanism and phagocytosis by alveolar macrophages will be faster because large particles do not allow the drug to penetrate the lungs quickly.^39^

## Inhalable Nanomedicine Platform for COPD and Preclinical and Clinical Evidence In vitro Studies

###  Liposome

 Liposomes are among the most extensively studied nanoparticle-based drug delivery systems for pulmonary applications, including COPD. Their phospholipid-based structure allows high biocompatibility with lung tissue and facilitates drug encapsulation for both hydrophilic and lipophilic compounds. Preclinical studies have shown that liposomes may enhance drug absorption across lung epithelial cells, improve permeability, and increase uptake by alveolar macrophages, thereby potentially improving local drug retention and therapeutic efficacy. In addition, liposomal formulations have been reported to prolong drug release and reduce pulmonary clearance, which may contribute to sustained therapeutic effects. Some inhalable liposomal products have progressed into clinical trials for other pulmonary conditions, indicating their translational potential. However, it is important to note that most available data specific to COPD remain limited to in vitro and animal studies. These experimental models may not fully capture the complexity of COPD pathophysiology in humans, including chronic inflammation, airway remodeling, and comorbid conditions. Furthermore, clinical evidence demonstrating clear therapeutic benefits of inhaled liposomes in COPD patients is still scarce. Therefore, while liposomes represent a promising delivery platform, their clinical applicability in COPD requires further validation through well-designed human studies.

###  Solid Lipid Nanoparticle (SLN) and Nanostructured Lipid Carrier (NLC)

 Solid Lipid Nanoparticles (SLNs) and Nanostructured Lipid Carriers (NLCs) are advanced lipid-based drug delivery systems developed as inhalable nanomedicine platforms for the treatment of CPOD. Both systems consist of a solid lipid core (in SLNs) or a mixture of solid and liquid lipids (in NLCs) stabilized by surfactants, forming a submicron structure with an average size of 100–300 nm, which is ideal for deposition in the lower respiratory tract. This size allows particle penetration into the alveoli, while the lipid structure provides high biocompatibility and the potential to increase drug retention time in the lung mucosa.^40^ NLCs were developed as an advanced generation of SLN to overcome the limitations of drug loading capacity and drug expulsion due to overly regular lipid crystallization in SLN.^41^ With the addition of a liquid lipid fraction, NLCs have a less regular matrix so that they can accommodate more lipophilic drug molecules and maintain system stability over a long period of time.

 The mechanism of drug release from SLNs and NLCs in inhalation systems is primarily controlled by diffusion and lipid degradation. In the early phase of deposition in the lungs, drugs are released from the particle surface through diffusion, which is influenced by concentration gradients and lipid matrix permeability. Subsequently, the slower release phase is controlled by lipid degradation by pulmonary lipase enzymes, resulting in a sustained release profile that prolongs drug exposure at the target site. This has been demonstrated in budesonide and curcumin-based NLC formulations, which show a threefold increase in the half-life of the drug in the lungs compared to conventional suspensions.^42^ Additionally, the lipid structure, which resembles endogenous pulmonary surfactant, enhances interaction with the mucus layer and allows deeper penetration through fusion or adsorptive endocytosis mechanisms in type II alveolar epithelial cells.

 In vitro and preclinical studies show that the use of SLN and NLC in inhalation systems can increase drug deposition efficiency and reduce local toxicity compared to conventional nebulization formulations. For example, an SLN formulation containing fluticasone propionate administered intratracheally showed a 250% increase in pulmonary bioavailability compared to a standard inhalation solution.^43^ Meanwhile, salbutamol sulfate-based NLCs show an increase in drug retention in lung tissue for up to 24 hours without mucosal irritation.^44^ Another advantage of this system is its ability to protect drugs from oxidative degradation due to oxygen exposure during nebulization, as well as to reduce the frequency of drug administration due to its prolonged release effect. Therefore, SLN and NLC are considered promising inhalation delivery systems in COPD therapy as they optimize drug targeting, enhance particle deposition efficiency, and minimize systemic effects commonly observed in conventional inhalation therapy.

###  Polymeric Nanoparticles

 Polymeric nanoparticles, particularly those based on materials such as PLGA, chitosan, and polyethylene glycol (PEG), have shown potential as inhalable drug delivery systems for respiratory diseases. These systems offer advantages including controlled drug release, enhanced stability, and improved interaction with the mucus layer, especially in the case of mucoadhesive polymers like chitosan. Several in vitro and in vivo studies have demonstrated that polymeric nanoparticles can enhance drug penetration through the mucus barrier, prolong retention time in the lungs, and reduce inflammatory responses. These properties are particularly relevant in COPD, where mucus hypersecretion and chronic inflammation are key pathological features. However, similar to other nanocarrier systems, the majority of evidence remains preclinical. The long-term safety of polymeric nanoparticles, including potential accumulation and immunogenicity, is not yet fully understood. Moreover, variability in formulation design and experimental conditions across studies makes it difficult to directly compare outcomes or predict clinical performance. Therefore, further research, particularly well-controlled clinical trials, is required to establish their role in COPD therapy.

###  Micelles, Dendrimers, Hybrid Carriers

 Micelles, dendrimers, and hybrid carriers are three major platforms in the development of nanotechnology-based drug delivery systems that are widely adopted for modern inhalation formulations. Micelles consist of amphiphilic molecules that spontaneously associate to form a spherical structure with a hydrophobic core and a hydrophilic outer layer. The micelle core functions as a place for encapsulating lipophilic compounds, while the hydrophilic layer, generally consisting of polyethylene glycol (PEG) chains, provides steric stability and increases particle diffusion on the surface of the respiratory tract mucus.^45^ Dendrimers, on the other hand, have a symmetrical branched macromolecular structure consisting of a core, tiered branches (generations), and reactive terminal groups that allow the conjugation of drugs, targeting ligands, and stabilizing compounds.^46^ The complexity of the dendrimer architecture provides high flexibility in charge, size, and branch density settings, enabling the system to carry both hydrophilic and lipophilic compounds simultaneously. Meanwhile, hybrid carriers combine the characteristics of lipid and polymeric materials in a core–shell or nano-in-micro structure, resulting in a system with high encapsulation capabilities like lipids, but with precise release control like polymers.^47^ These three systems are designed to produce particles with an ideal mass median aerodynamic diameter (MMAD) of 1–5 µm, enabling effective deposition in the bronchioles and alveoli after conversion to aerosol form through spray-drying or nebulization.^27^

 The drug release mechanism in these three systems depends on complex interactions between the composition of the carrier material, the pulmonary environment, and the physiological conditions of the alveolar fluid. In the micelle system, drug release begins with the desorption of molecules adsorbed on the surface due to concentration gradients, followed by the diffusion of active compounds from the hydrophobic core to the local aqueous phase. This mechanism can be modulated by adjusting the length of the copolymer block or adding pH- or enzyme-sensitive bonds, so that release occurs gradually and in a controlled manner. For example, pH-sensitive micelles made from poly(methacrylic acid-co-ethyl acrylate) can undergo disintegration at acidic pH due to protonation of the carboxylate group, accelerating drug release.^48^ In dendrimer systems, drug release generally occurs through two main mechanisms, namely the cleavage of chemical bonds in the terminal groups conjugated with the drug (cleavage-triggered release) and the diffusion of compounds from the internal pores of the dendrimer (pore-mediated diffusion). Enzymatic reactions involving pulmonary esterases or proteases can accelerate the cleavage of ester or peptide bonds used as linkers, resulting in selective release in the target tissue.^49^ In hybrid carrier systems, drug release occurs in two phases: rapid release (burst release) from the outer polymeric layer, which dissolves quickly, followed by slow release (sustained release) from the lipid core, which degrades gradually. In the context of aerosolization, lipid-polymer-based nano-in-micro particles containing leucine as a dispersion agent are capable of exhibiting a fine particle fraction (FPF) above 60% with a sustained release efficiency of up to 80% over 48 hours.^47^

 Micelles, dendrimers, and hybrid carriers offer various advantages over conventional solution- or suspension-based inhalation systems. All three are capable of improving the stability of compounds that are easily degraded by oxidation or lung enzymes, prolonging local retention time, and enabling the delivery of combinations of drugs with different solubilities.^50^ Micelles offer advantages in mucus penetration because their PEG layer reduces interactions with mucus glycoproteins, while dendrimers enable specific targeting through modification of terminal groups with ligands such as hyaluronic acid or epithelial receptor peptides.^51^ Hybrid carrier systems are promising for long-term therapy, as they enable rapid initial drug release followed by sustained maintenance of therapeutic concentrations. However, there are a number of important challenges that need to be overcome before clinical application can be carried out, including the potential for chronic toxicity due to the accumulation of non-biodegradable polymers, complex interactions with alveolar surfactants, and the possibility of changes in the release profile due to the formation of a protein corona. Therefore, systemic evaluation via air–liquid interface (ALI) studies, bronchoalveolar lavage fluid (BALF) testing, and cascade impaction analysis for MMAD and emitted dose measurements are crucial steps in assessing the performance and safety of this system. Formulation optimization through a spray-drying approach with excipients such as mannitol and leucine, accompanied by post-aerosolization stability testing, represent the most promising strategy to ensure the long-term effectiveness of inhalable nanomedicines based on micelles, dendrimers, and hybrid carriers.^52^ The characteristics, representative drugs, and key performance of the major nanocarrier systems used for pulmonary drug delivery are summarized in Table 1.

**Table 1:** Characteristics and Performance of Various Nanocarriers for Drug Delivery Systems

**Type of nanocarrier**	**Drugs / Contained agents**	**Performance / Key findings**	**References**
Liposomes	Curcumin	Increases uptake by lung cells, improves epithelial permeability, prolongs drug half-life by ± 10 × and AUC by thousands of times compared to oral administration; high inhalation efficacy but clinical data on COPD is still limited.	^53–55^
Micelles	Common lipophilic compounds, PEGylated micelles	The amphiphilic structure facilitates the encapsulation of lipophilic drugs; mucus penetration is increased due to steric stabilization; drug release occurs through desorption and diffusion; can be modified to be pH-sensitive/enzyme-responsive.	^48,49^
Dendrimer	Hydrophilic & lipophilic (various molecules), conjugated drugs	The branched structure allows for high loading, specific targeting via terminal ligands; release via chemical bond cleavage (enzyme-triggered) or diffusion from dendrimer pores.	^13^
Hybrid carriers (lipid–polymer, nano-in-micro)	Various combination inhalation medications	Combines the advantages of lipids (high encapsulation) & polymers (controlled release); produces an ideal MMAD of 1–5 µm; FPF > 60% and gradual release of up to 80%/48 hours.	^9,24^
SLN (Solid Lipid Nanoparticle)	Fluticasone, budesonide, curcumin	Solid lipid core, size 100–300 nm; increases pulmonary bioavailability (up to 250%), depresses toxicity; drug release through initial diffusion and subsequent lipid degradation.	^40,43^
NLC (Nanostructured Lipid Carrier)	Salbutamol, budesonide, curcumin	Solid + liquid lipid matrix increases load capacity and stability; extends drug retention up to 24 hours; better mucus penetration, does not cause irritation.	^41^
Polymeric nanoparticles (PLGA, chitosan, PEG)	Various anti-inflammatory and antioxidant drugs	PLGA provides controlled release, chitosan is mucoadhesive so it can increase mucus penetration, reduce inflammation and oxidative stress, and increase drug retention in the lungs in animal models.	^56^

## Advantages Over Conventional Inhalation Therapy

 Inhalation-based nanomedicine offers a number of advantages over conventional inhalation therapy in the management of lung diseases, including COPD. Through the use of nanoparticle carriers such as liposomes, PLGA, and chitosan, this system is able to improve treatment effectiveness by improving drug distribution in lung tissue, regulating drug release in a more controlled manner, and reducing the occurrence of systemic side effects. Some key advantages:^52^

▪ Targeted lung delivery: higher local doses or lower systemic effects.
▪ Controlled and prolonged drug release: reduced dosing frequency.
▪ Increased lipophilic drug solubility and local bioavailability.
▪ Improved mucus penetration/retention in the lungs (mucoadhesive carriers such as chitosan).
▪ Potential to reduce systemic side effects and lower total therapeutic dose.

## Translational Challenges to Clinical Application

###  Long-Term Safety and Stability

 Long-term safety remains one of the major barriers in developing inhalable nanomedicine, as repeated administration to the lungs may trigger chronic adverse effects.^57^ Although biodegradable materials such as PLGA are considered biocompatible, their degradation rate in the lungs may influence drug release, leave residual by-products, and induce inflammation that can potentially progress into oxidative stress or fibrosis.^58^ In addition, several nanoparticles, including carbon nanomaterials and inorganic particles, have been shown to elicit oxidative and inflammatory responses under chronic exposure. Delivery systems based on synthetic materials also carry the risk of immunogenicity, namely the formation of antibodies that may reduce therapeutic effectiveness.^59^ The physicochemical characteristics of nanoparticles greatly determine their interactions with pulmonary tissues; therefore, safety assessments must be conducted under repeated-dose exposure and in disease-relevant models, such as chronic cigarette smoke exposure in COPD. Through such studies, biodistribution, clearance mechanisms, biodegradation, and immunogenic potential can be evaluated.^60^

 From a formulation perspective, physicochemical stability also poses a significant challenge. Nanoparticles are prone to aggregation during processes such as spray drying or lyophilization, as well as during storage due to humidity and temperature, which can affect particle size, respirable fraction, and dose consistency.^61^ Techniques such as spray drying can help generate inhalable powders, but process conditions must be optimized to prevent degradation of the active ingredient.^62^ Long-term stability studies for inhalable nanomedicine remain limited, and their outcomes vary widely across dosage forms and drying methods. Therefore, future research needs to emphasize specialized stability testing protocols for submicron particles, the use of excipients that are safe for inhalation, and data that can bridge laboratory-scale conditions with commercial storage environments.^63^

###  Clinical Failure Risk

 Despite promising outcomes in preclinical studies, the successful clinical translation of inhalable nanomedicine remains limited with a substantial proportion of candidates failing during clinical trials. Analyses of nanomedicine development indicate that, although early-phase trials often demonstrate acceptable safety profiles, the success rate decreases markedly in later stages of clinical testing. For instance, the success rate of inhalable nanomedicine candidates reported in Phase I trials can exceed 90%, but it drops to approximately 48% in Phase II and further declines to around 14% in Phase III trials, primarily due to insufficient therapeutic efficacy rather than toxicity.^64^

###  Large-Scale Manufacturing & GMP Standards

 The large-scale production of inhalable nanomedicine remains a major challenge in the translational pathway from research to industry. Many laboratory-based methods such as batch nanoprecipitation, emulsification, or sonication cannot be directly scaled up because it is difficult to maintain consistent particle size, distribution, and drug loading across batches.^65^ At the industrial level, processes such as spray drying or freeze drying also require strict control of temperature, pressure, and drying rate to ensure that the particle structure remains stable without causing degradation of the active ingredient.^66^ From a regulatory standpoint, the implementation of cGMP standards for inhalable nanomedicine still faces limitations because several critical parameters such as CQA and CPP do not yet have globally harmonized standards. Therefore, QbD and Process Analytical Technology (PAT) approaches are increasingly recommended to maintain quality consistency during scale-up.

 Additional challenges include the validation of analytical methods specific to nanomaterials, control of residual solvents, and ensuring process cleanliness for inhalation products. The transition from pilot to commercial scale also requires well-designed scale-up strategies, as changes in equipment or process parameters can affect aerosol performance, pulmonary deposition efficiency, and bioavailability. On the other hand, economic data and commercialization prospects for inhalable nanomedicine remain limited, making industry stakeholders more cautious about investment.^18^ Therefore, more cross-batch comparison studies, process validation, and comprehensive risk assessments are needed to bridge the gap between academic research and industrial manufacturing.

## Regulatory, Accessibility, Cost, and Patient Adherence Considerations

 The translational challenges of inhalable nanomedicine extend beyond technical and manufacturing aspects, encompassing issues related to regulation, production costs, and patient adherence. From a regulatory perspective, there are still no international guidelines specifically dedicated to inhalation nanomedicine. Agencies such as the EMA and FDA continue to rely on general frameworks for conventional drug products, while the unique characteristics of nanomaterials, such as particle size, surface charge, and biodistribution behavior require additional evaluation parameters that have yet to be standardized. Differences in regulatory approaches between countries also slow global harmonization of approvals and may hinder product commercialization.^18^

 In addition, the complexity of the manufacturing process and the need for Good Manufacturing Practice (GMP) facilities with stringent particle control make the development and production costs of nanomedicine significantly higher than those of conventional inhalation therapies.^67^ High costs may limit patient access, particularly in developing countries where the burden of COPD is disproportionately higher.^12^ Although nanoparticle-based delivery systems have been shown to improve efficacy, these novel dosage forms may require additional user training and can reduce adherence if not designed ergonomically.

## Innovation for Further Research

 The strategy of combining anti-inflammatory and antioxidant agents into a single inhaled carrier aims to simultaneously target two key pathogenetic mechanisms of COPD: chronic inflammation and oxidative stress. Nanoparticle or nano-in-micro dry powder formulations encapsulating this combination can increase drug residence time in the lungs and provide controlled release, reducing systemic exposure.^68^ Several studies have shown that combining corticosteroids or inflammatory inhibitors with antioxidants such as N-acetylcysteine or edaravone significantly reduces ROS and proinflammatory cytokine levels in animal models of COPD.^69^ Although preclinical results are promising, further studies are needed to determine chronic lung toxicity and optimize the drug-loading ratio before clinical translation.^70^

 The integration of inhaled nanomedicine formulations with smart inhaler devices is an innovative step to improve therapy effectiveness through real-time patient adherence monitoring. Modern smart inhalers can record usage time, inspiratory flow, and provide automatic reminders to improve adherence.^71^ The obtained data can be combined with the nanoparticle pharmacokinetic profile to personalize dosage adjustments. A preliminary study showed that digital inhaler technology can improve usage techniques and pulmonary outcomes, although evidence for reduced exacerbations is still emerging.^69^ The synergy between digital sensors and precise nanoformulations is expected to form the basis for the concept of an adaptive and individualized smart inhaler.

 The development of personalized medicine is driving the management of COPD based on individual phenotypes and biomarkers such as eosinophil counts, inflammatory patterns, and oxidative profiles. In the context of nanomedicine, this opens up opportunities to tailor drug payloads and delivery systems to patient profiles to enhance therapeutic response.^72^ For example, patients with predominantly oxidative stress may benefit from antioxidant-based carriers, while those with an eosinophilic phenotype could be targeted with anti-eosinophilic delivery systems. This approach requires the integration of clinical data, biomarkers, and digital inhaler data to generate truly individualized therapy algorithms.^71^ The key challenges going forward are the accessibility of biomarker validation and the availability of adequate diagnostic infrastructure for widespread implementation.

 Efforts to accelerate the translation of inhaled nanomedicines into clinical practice require a comprehensive, multidisciplinary framework. The “DELIVER” framework emphasizes the importance of translational planning from the formulation design stage, including aspects of GMP manufacturing, long-term safety, and early dialogue with regulators. Furthermore, recent translational studies suggest the use of chronic lung organ-on-chip and animal models to more predictively assess the safety of inhaled nanoparticles.^70^ The integration of biomarker endpoints, digital inhaler data, and precision medicine-based clinical trials is key to accelerating the introduction of inhaled nanomedicine innovations into COPD therapy.^72^

## Conclusion

 Inhalable nanomedicine offers a promising advancement for improving COPD therapy by enhancing drug deposition in the lungs, increasing local bioavailability, and reducing systemic side effects compared with conventional inhalation treatments. Various nanocarriers such as liposomes, SLN/NLC, polymeric nanoparticles, and hybrid systems have demonstrated improved therapeutic performance in preclinical studies through controlled release and better targeting of diseased lung tissue. However, major challenges remain, including long-term safety concerns, formulation stability, high manufacturing complexity, and the absence of standardized regulatory guidelines. Therefore, further clinical studies, advanced translational strategies, and multidisciplinary collaboration are needed to ensure the safe, effective, and scalable implementation of inhalable nanomedicine for COPD management in the future.

## Competing Interests

 The authors unequivocally state that they have no conflicts of interest.

## Ethical Approval

 Not applicable.
